# Bioinformatic Characterization of Sulfotransferase Provides New Insights for the Exploitation of Sulfated Polysaccharides in *Caulerpa*

**DOI:** 10.3390/ijms21186681

**Published:** 2020-09-12

**Authors:** Simone Landi, Sergio Esposito

**Affiliations:** Department of Biology, University of Naples “Federico II”, Via Cinthia, I-80126 Napoli, Italy; simone.landi@unina.it

**Keywords:** sulfatase, *Caulerpa lentillifera*, antioxidants, anti-inflammatory molecules, sulfation, *Caulerpa taxifolia*, galactomannan

## Abstract

*Caulerpa* is an unusual algal genus from *Caulerpaceae* (Chlorophyta, Bryopsidales). Species from this family produce a wide range of metabolites suitable for biotechnology applications. Among these, sulfated polysaccharides (SPs) are often highly desirable for pharmaceutical and nutraceutical applications. Here, we provide a classification of sulfotransferases from *Caulerpa*; these important enzymes catalyze the nodal step for the biosynthesis of SPs. For this, we performed phylogenetic, genomic, expression analyses and prediction of the protein structure on sulfotransferases from *Caulerpa*. Sequences, domains and structures of sulfotransferases generally shared common characteristics with other plants and algae. However, we found an extensive duplication of sulfotransferase gene family, which is unique among the green algae. Expression analysis revealed specific transcript abundance in the pinnae and rachis of the alga. The unique genomic features could be utilized for the production of complex SPs, which require multiple and specific sulfation reactions. The expansion of this gene family in Caulerpaceae would have resulted in a number of proteins characterizing the unique SPs found in these algae. We provide a putative biosynthetic pathway of SPs, indicating the unique characteristics of this pathway in *Caulerpa* species. These data may help in the future selection of *Caulerpa* species for both commercial applications and genetic studies to improve the synthesis of valuable products from *Caulerpa*.

## 1. Introduction

*Caulerpa* species (*Caulerpaceae* - Order Bryopsidales - Chlorophyta) are composed solely of huge, multinucleated, single siphonous cells. *Caulerpa* species are considered to produce the biggest single cells of any living organism on Earth [1,2]. *Caulerpa* species are of economic interest as they are often both highly invasive in marine ecosystems and of increasing biotechnological value [3,4,5]. The presence of *Caulerpa* species is particularly problematic in the Mediterranean region, where the Australian *Caulerpa cylindracea* is now widespread and widely invasive in the ecosystem [3,6] causing serious problems for the ecology of the Mediterranean Sea [6,7]. 

In addition to ecological problems, invasive *Caulerpa* species often cause economic problems for commercial fishery. In the southern Mediterranean Sea, *Diplodus sargus* (seabream) is particularly affected by *Caulerpa*, representing a primary food source [8]. Due to the presence of high levels of the bisindole alkaloid called caulerpin [9], *Diplodus sargus* fed with *Caulerpa* presents an alteration of lipids, polyunsaturated fatty acid (PUFA) and ω-3 content which may be detrimental to fish growth rates and development [10,11,12].

Despite their potential for invasion, *Caulerpa* is often used for human consumption, and is named “green caviar” or “sea grape” in countries such as Japan, China and the Pacific islands [5]. Indeed, in Fiji, Samoa and Tonga the market value reached by the harvested alga is estimated about USD 270,000 per year resulting in a production of over 120 tonnes of fresh *Caulerpa*, which plays a crucial role in the economy of these countries [13]. In recent years, *Caulerpa* species may be highly economically useful as they can be readily used for the production of bio-compounds for nutraceutical, pharmaceutical and cosmetic purposes [4,5,14,15,16,17]. *Caulerpa* produces interesting metabolites, namely caulerpin, sulfated polysaccharides (SP), racemosin, alkaloids, xyloglucans and fatty acid derived products, which are highly desirable for commercial purposes, and are not fully commercially exploited yet [4,5]. Among these, SPs showed a remarkable pharmaceutical potential, making this class of molecule one of the most interesting compounds biosynthesized by *Caulerpa* [18,19,20]. SPs can be synthesized by different biosynthetic pathways, depending on the polysaccharide backbone [21]. In general, algae synthesize a wide range of different SPs depending on their taxonomy. For example, sulfated fucans in brown algae, sulfated galactans in green and red algae and carrageenan in red algae [22]. However, little is known about SP production in *Caulerpa*.

The key enzymatic step for the biosynthesis of SP is catalyzed by sulfotransferase [21,22]. This class of enzymes catalyzes sulfation reaction where a sulfuryl group (SO_3_^−^) from 3-phosphoadenosine-5-phosphosulfate (PAPS) is transferred to a hydroxyl group of an acceptor [23,24]. Polysaccharides are important components of seaweed biomass, playing a wide range of activities, particularly when containing functional groups as sulfates [25]. This modification affects polysaccharide properties, regulating solubility, charge conformation and increasing immune-stimulatory activity responses [19]. As shown in Table 1, *Caulerpa* extracts, or purified compounds, showed potential beneficial properties. Although some of these reports may be too optimistic in their conclusions, the effective potentiality of *Caulerpa* still remains to be explored. Several authors reported antioxidant and anti-inflammatory effects; these activities were reported both for *Caulerpa* extracts [26,27,28,29] and purified SPs [19,20,30,31].

A potential application of the antioxidant ability of *Caulerpa* SPs was recently reported [4,20,27], showing promising results regarding the treatment of calcium oxalate crystals in antiurolithic cures [20]. Furthermore, promising immunostimulatory effects were observed using a different class of polysaccharides from *Caulerpa* species [32,33,34]. Recently, four different novel xylogalactomannans were identified [19,33]; particularly, CLGP4 showed the higher sulfate content (21.26% dry weight) and the in-vitro stimulation of macrophages by increasing proliferation, phagocytosis and production of nitric oxide (NO) and phosphatase activity [33]. Moreover, this SP showed beneficial effects to the HT29 carcinoma cells inducing a reduced synthesis of IL-1b, TNF-a, SIgA and mucin2 [19]. Similar concentrations were found testing four SPs from *Caulerpa cupressoides*, composed by galactose, glucose, mannose, rhamnose and xylose in different molar ratios [31]. These SPs showed beneficial effects both on murine RAW 264.7 macrophages by increasing cell mobility and nitric oxide and cytokine production both reducing pain in the temporomandibular joint of rates [30,34]. Similar effects were reported using lectin fractions from the same species [35]. Consistently, ethanolic extract from *Caulerpa okamurae* reduced the molecular expression of TNF-α, interleukin-6 and monocyte chemoattractant in RAW 264.7 macrophages [26]. Immunomodulatory effects were also identified in a novel soluble polysaccharide, called CRVP-1, from *Caulerpa racemosa* var *peltata* [32]. CRVP-1 consisted of a backbone of α-D-Mannose units linked with side chain of β-D-Galactose units and sulfate residues; its administration to macrophages induced an increased secretion of cytokines and production of NO [32]. An interesting gastroprotective role against ethanol damages has been suggested using SPs from *Caulerpa mexicana*. The administration of extracts from this *Caulerpa* species to mice with gastric damage induced normalized levels of glutathione and thiobarbituric acid together with an increased involvement of prostaglandins [36]. Furthermore, a collection of SPs was purified from Brazilian *Caulerpa prolifera*, showing a sulfate/total sugar ratio from 0.03 to 0.44 [37]. Among these, CP0.5 showed the major amount of sulfate and osteogenic induction potential on human mesenchymal stem cells, increasing alkaline phosphatase activity and calcium accumulation [37].

Hence, despite there being a plethora of studies on potential pharmaceutical properties of members of *Caulerpa*, to our knowledge, no bioinformatic, biochemical or phylogenetic comprehensive studies have been performed so far on key enzymes of the pathways of this secondary metabolism of *Caulerpa*. Particularly, there is no description regarding the complexity and unique characteristics of the SP biosynthetic pathway in *Caulerpa*. The aim of this paper is to develop an integrated bioinformatic, phylogenetic and expression analysis approach to provide a full characterization of the sulfotransferase of *Caulerpa*. Furthermore, a general and updated overview of the beneficial effects of *Caulerpa* biomolecules is provided.

## 2. Results

### 2.1. Caulerpa Showed an Unconventional Number of Sulfotransferase

In order to characterize SPs biosynthesis in *Caulerpa sp*., and identify the enzymes responsible for sulfation of carbohydrates, a bioinformatic approach was carried out to identify the various isoforms of sulfotransferase and sulfatase.

Different putative genes were found in *Caulerpa lentillifera* genome scanning using the OIST (Okinawa Institute of Science and Technology) marine genomic database (https://marinegenomics.oist.jp/umibudo/search/index?project_id = 55).

In order to do this, five different PFAM domains were selected: PF00685, PF03567, PF13469, PF06990 and PF00884. PF00685 represents the sulfotransfer-1 PFAM domain characterized by the PAPS binding site and by the interPro accession IPR000863 [46,47]. All human carbohydrate sulfotransferases from group 1 to group 7 contain this PFAM domain as well as flavoyl-, aryl-, alcohol- and phenol-sulfotransferases [48]; PF03567 is identified by the interPro accession IPR005331, representing the sulfotransferases able to transfer sulfate to position 3,4 and 6 of carbohydrate groups in glycolproteins and glycolipids [47,48]; PF13469 represents the sulfotransfer-3 PFAM domain, specifically belonging to the algal lineage [46]; PF06990 represents the galactose sulfotransferase domain identified by the interPro accession IPR009729 [46,47]; PF00884 characterizes the sulfatase PFAM domain and the intePro accession IPR000917 [47].

In total, 46 transcripts, containing one of this PFAM domain and coding for putative sulfotransferase related proteins were identified (Table 2). PF00685 and PF13469 showed redundant hit. *Caulerpa* showed no PF06990, which is considered an algal specific sulfotransferase domain [46]. Significance and domain position are indicated in Supplemental Table S1.

Different alternative pairs of transcripts were identified: g1631.t1-2, g5902.t1-2 and g4272.t1-2; the alignments among these transcripts were shown in Supplementary Material (Figure S1–S3).

PFAM sulfotransferase domains PF09037, PF05935 and PF14269 were described as poorly represented in plants and algae; our data report no identified hits and, therefore, these domains have been excluded in Table 2 [46]. BLASTp approaches were performed using each identified sulfotransferase from *Caulerpa* to validate their functions. This analysis reported six transcripts with different functions: g1262.t1 (Glutamyl endopeptidase), g3147.t1 (Peptidyl-prolyl cis-trans isomerase), g395.t1 (ABC transporter), g3703.t1 (Acetyl-CoA carboxylase), g3783.t1 (Serine acetyltransferase) and g4170.t1 (Cytochrome p450). These transcripts were excluded in the following analyses. The number of the identified transcripts is in agreement with Arimoto et al., [1], reporting 40 different genes ascribed to the onthology category “Sulfotransferase activity” (GO:0008146). This is higher in the *Caulerpa* genome compared with *Chlamydomonas reinhardtii*, *Volvox carteri*, *Chlorella variabilis* and *Ostreococcus tauri* genomes, which showed 14, 17, 14 and 9 GO:0008146, respectively [1]. We found that the *C*. *lentillifera* genome showed a higher number of genes compared with other green algae, suggesting a specific genome expansion.

**Figure 1:**
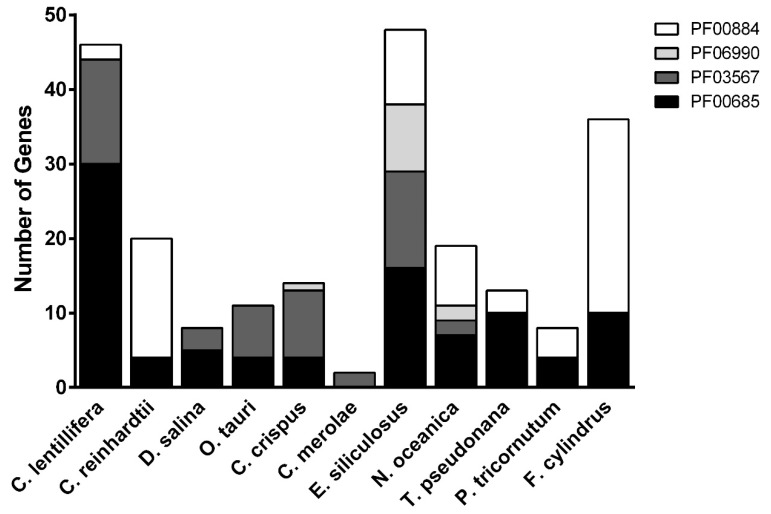
Number of genes containing sulfotransferase related PFAM domains (PF00685—black, PF03567—dark grey, PF06990—light grey and PF0084—white) in different algae species. Legend and classification (Abbr., scientific name Order, Class *Phylum): *C*. *lentillifera*, *Caulerpa lentillifera* (Ulvophyceae, Bryopsidales); *C*. *reinhardtii*, *Chlamydomonas reinhardtii* (Chlorophyceae, Chlamydomonadales); *D*. *salina*, *Dunaliella salina* (Chlorophyceae, Volvocales); *O*. *tauri*, *Ostreococcus tauri* (Mamiellophyceae, Mamiellales); *C*. *crispus*, *Chondrus crispus* (Rhodophyta*, Florideophyceae); *C*. *merolae*, *Cyanidioschyzon merolae* (Cyanidiophyceae, Cyanidiales); *E*. *siliculosus*, *Ectocarpus siliculosus* (Phaeophyceae, Ectocarpales); *N*. *oceanica*, *Nannochloropsis oceanica* (Eustigmatophyceae, Eustigmatales); *T*. *pseudonana*, *Thalassiosira pseudonana* (Coscinodiscophyceae, Thalassiosirales); *P*. *triconutum*, *Phaeodactylum tricornutum* (Bacillariophyceae, Bacillariales); *F*. *cylindrus*, *Fragilariopsis cylindrus* (Bacillariophyceae, Bacillariales).

Gene duplication occurrence was reported for different enzymatic families such as ubiquitin-related proteins, peptidase and peroxidase [1]. In order to compare *Caulerpa* sulfotransferase to the whole algal lineage, a number of genomes were mined to identify those genes containing sulfotransferase-related PFAM domains. As shown in Figure 1, the brown algae *Ectocarpus siliculosus* and the diatom *Fragilariopsis cylindrus* are the only two species showing a comparable number of putative sulfotransferases with *C*. *lentillifera*. According to Ho [48], *C*. *merolae* (*Cyanidiales*, Rhodophyta) showed a limited number of sulfotransferases.

We compared genome size and number of genes found here with different algae. *Caulerpa* has a 28 Mb genome and 9311 genes coding for proteins, displaying a gene density about 3.2 gene/kb while *Chlamydomonas* showed a 111.1 Mb genome with 17141 genes coding for proteins; therefore, *Caulerpaceae* show a double gene density with respect to the model green alga [1,49]. This evidence suggests a possible large duplication for sulfotransferase genes for *Caulerpa*. On the other hand, the distribution of the various PFAM domains is different comparing the species with a high number of sulfotransferases: *C*. *lentillifera* showed 30 proteins with a PF00685 domain, *E*. *siliculosus* (*Phaeophyceae*) exhibited an homogenous distribution of PFAM domains and *F*. *cylindrus* (*Bacillariophyceae*) shows 26 proteins with a PF0084 domain. Brown algae have a unique cell wall composed by both common polysaccharides and unique compounds. *In Phaeophyceae* an important role is played by a specific class of SPs named Fucoidans [50]. Among the *E*. *siliculosus* sulfotransferases, a high number was identified to be related to polysaccharides reflecting the needs of multiple sulfation reactions required for fucoidans biosynthesis [51]. Similar complex cross-reactions could be required for an adequate sulfation of polysaccharides in *Caulerpa*.

### 2.2. Phylogenetic Characterization of Caulerpa Sulfotransferase

SPs represent a major and ancestral component present in the whole algal lineage. It was estimated that both SPs and β-1-3-glycans were the original components of the cell wall of the last plant and algal eukaryotic common ancestor [51]. SPs biosynthetic pathway required a sulfation step along the carbohydrate backbone, which is catalyzed by different types of sulfotransferases.

The sulfotransferase family is composed of different groups: carbohydrate sulfotransferase (CHSTs) and formylglycine-dependent sulfatase (FGly-SULF) [22]. CHSTs—the main class of enzymes involved in the synthesis of SPs—are particularly represented in algae, microalgae and diatoms, underlying the importance of these compounds in cell walls, and their roles in regulation processes [21,52]. CHSTs should be divided in two different families based on the presence of conserved domains [22]. FGly-SULF are able to use many different substrates, namely glucosinolates, steroids, glycosaminoglycans, proteoglycans, glycolipids and others [23].

**Figure 2:**
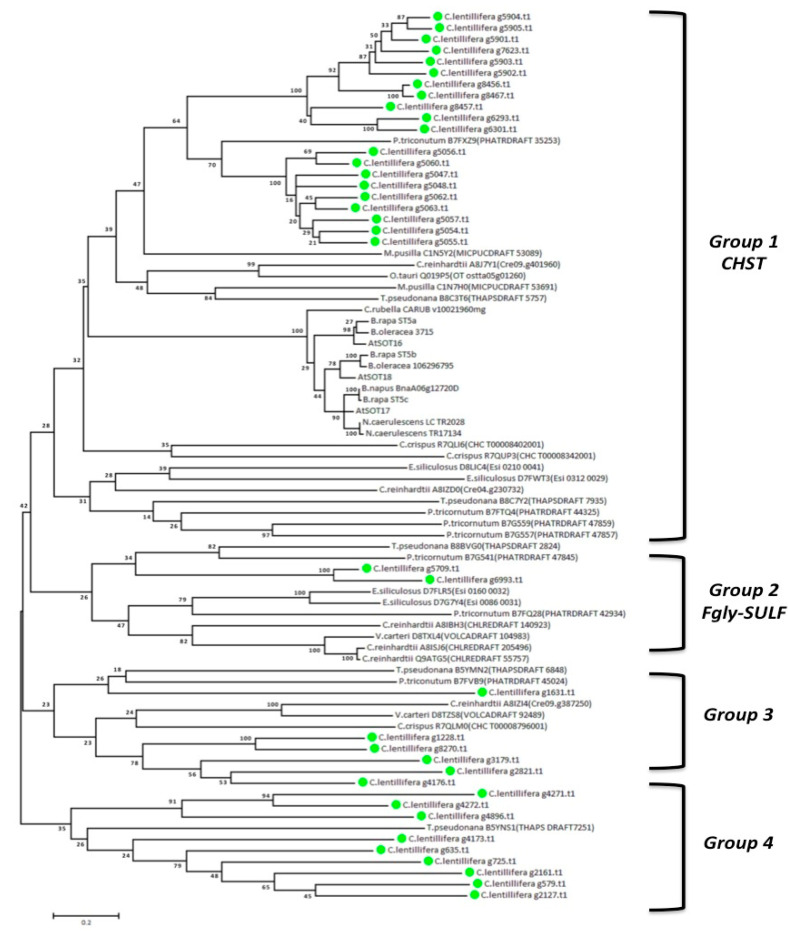
Un-rooted phylogenetic tree of putative sulfotransferase aminoacidic sequences constructed using maximum likelihood method. The bootstrapping test (replicate = 100) is indicated on each node, in order to verify the phylogeny. *Caulerpa lentillifera* sequences are highlighted by green circles.

To assign sulfotransferase from *Caulerpa* to various sub-families, a comparison in the amino acidic sequences of the 37 single transcripts was performed versus various known sulfotransferase from different algae, microalgae, diatoms and plants. Among these, we selected *Chlamydomonas reinhardtii*, *Volvox carteri*, *Micromonas pusilla*, *Ostreococcus tauri*, *Thalassiosira pseudonana*, *Phaeodactylum tricornutum*, *Ectocarpus siliculosus*, *Chondrus crispus*, *Arabidopsis thaliana*, *Capsella rubella*, *Brassica rapa* and *Brassica oleracea*. After a model-selection analysis, an un-rooted tree was constructed using the maximum likelihood method, in order to investigate the phylogenetic relationship (Figure 2).

The phylogenetic tree revealed the clustering of sequences in four major groups. A major cluster 1 included both CHST sulf 1 and 2 groups, containing 20 sequences and second FGly-SULF group containing 2 *Caulerpa* sequences. The third and fourth cluster showed the ambiguous presence of CHST sulf 1-2 and FGly-SULF sequences. This group contains 15 *C*. *lentillifera* sequences.

Unexpectedly, *Caulerpa* sequences often clustered together with sequences from the diatom *T*. *pseudonana* (Thapsdraft_7251, Thapsdraft_6848, Thapsdraft_2824) and *P*. *tricornutum* (Phatdraf_35253, Phatdraf_45024, Phatdraf_47845). The last three genes showed a down-regulation in *P*. *tricornutum* under nutritional starvation [53]. Diatoms sulfotransferases can be divided in two main groups, the first similar to human and cyanobacteria, and the other related to plants and algae [52]. As expected, Phatdraf_35253 is near to the *Caulerpa* sulfotransferases, fitting to the second group. Furthermore two *E*. *siliculosus* sulfotransferases, namely Esi0210_0041 and Esi0312_0029, were reported to be related to animal carbohydrate sulfotransferases [51]. These two proteins, together with other four sulfotransferases, were suggested as the best suitable candidates for the sulfation of glycosaminoglycans in brown algae [51].

### 2.3. Caulerpa Sulfotransferase Structures: Peculiarities and Similarities vs. Algae and Plants

**Figure 3:**
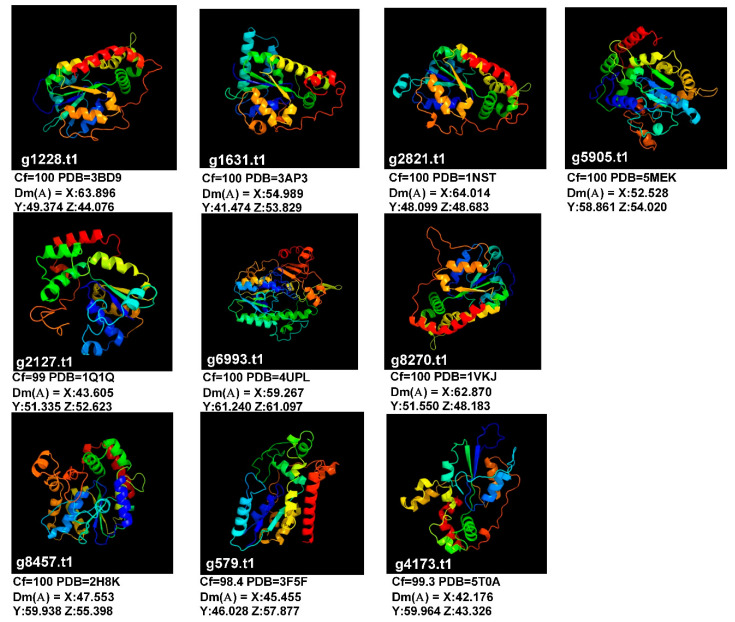
The 3D-structures of representative sulfotransferase from *C*. *lentillifera*. Further details in Table 3. Legend: g1228.t1 = Heparan sulfate glucosamine 3-o-sulfotransferase; g1631.t1 = Protein-tyrosine sulfotransferase 2; g2821.t1 = Heparan sulfate n-deacetylase/n-sulfotransferase; g5905.t1 = Sulphotransferase-18 from Arabidopsis; g8270.t1 = Haparan sulfate n-deacetylase/n-2 sulfotransferase; g8457.t1 = Human sulfotranferase sult1c3 in complex with pap; g579.t1 = Maltose-binding periplasmic protein, heparan sulfate 2-o; g4173.t1 = Maltose binding protein - heparan sulfate 6-o; g2127.t1 = Human pregnenolone sulfotransferase; g6993.t1 = N-acetylgalactosamine-6-sulfatase. Cf = Confidence; PDB = Protein data bank database ID; Dm = Dimension in angstrom.

A structure analysis of the *Caulerpa* sulfotransferase was made by using Phyre2 online software (Figure 3 and Table 3). As showed in Table 3, putative substrates were identified for a number of proteins. In total, 15 proteins related to sulfation of heparan sulfate, maltose, glucosamine and N-acetylgalactosamine were recognized. Moreover, the structure predictions of g1631.t1, g2127.t1 and g2161.t1 suggest no or minor roles about the sulfation of polisaccharides.

**Figure 4:**
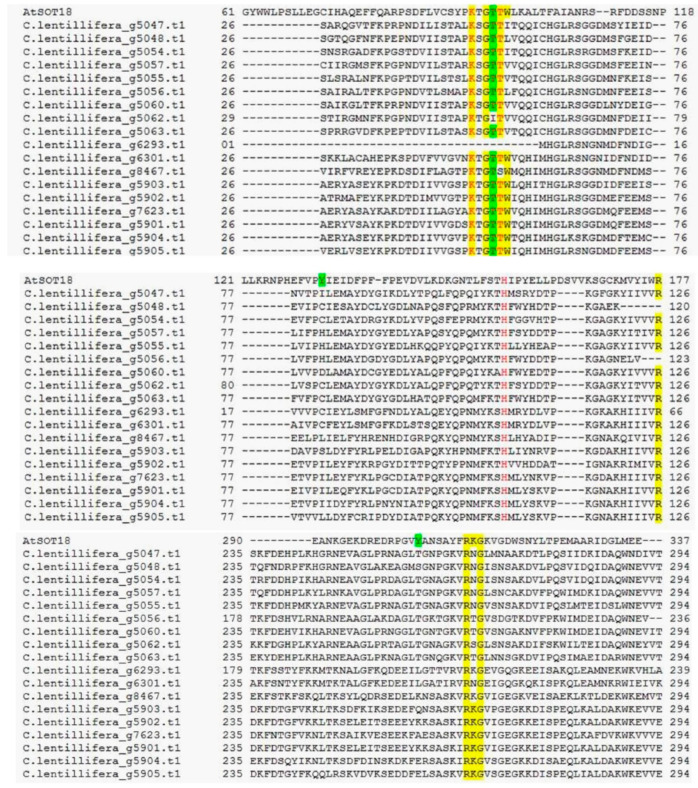
Alignment of *At*SOT18 to similar *C*. *lentillifera* protein sequences. Yellow highlighted residues indicate PAPS binding domain, green highlighted residues indicate sinigrin binding domain (for *At*SOT18). Catalytic domain residues Lys93, Threo97 and His155 were in red [46].

A high number of proteins showed a structure similarity with the sulphotransferase-18 from *Arabidopsis thaliana* (*At*1G74090—*At*SOT18). These proteins, together with *At*SOT16 and *At*SOT17, play a central role in plant glucosinolate metabolism [47]. Particularly, *At*SOT18 showed substrate specificity for long-chain desulfo-glucosinolate, 7-methylthioheptyl and 8-methylthiooctyl, all derived from methionine [54]. Recently, the structure of this protein was elucidated identifying key amino acids residues [55]. The alignment of *At*SOT18 and the related *C*. *lentillifera* sequences is shown in Figure 4. Critical residues involved in PAPS binding have been identified in *At*SO18 as Lys93, Gly95, Thr97, Arg177, Arg313, Lys314 and Gly315 (yellow highlighted). These residues were recurrently retrieved in *C*. *lentillifera* sequences as well (Figure 4). Similarly, other important residues, Lys243 and Phe285, have been identified in most of the analyzed sequences, while Cys283 was replaced by Ser in all *Caulerpa* sequence (not shown). Similarly, amino acids of the catalytic domain were conserved in *C*. *lentillifera* protein sequences; among these, His155 plays a critical role for the sulfation reaction in all sulfotransferases [46].

As expected, sugars and glucosinolates binding amino acid residues (e.g., sinigrin) were not conserved in *Caulerpa*, thus showing different substrates specificity for algal carbohydrates. Tyr130 and Tyr306 were replaced by different amino acids in *Caulerpa*. In *At*SOT18, the hydroxyl group of Tyr306 creates a hydrogen bond with oxygen in the glucopyranose ring of the sinigrin [55]. These amino acids are recurrently replaced in *Caulerpa* by cysteine, isoleucine and histidine. Furthermore, Tyr130, Thr96 and His155 stabilize the sulfate moiety by hydrogen bonds [55]. In a group of *Caulerpa* sulfotransferases, Tyr130 is replaced by threonine and serine, possibly playing a similar role.

**Figure 5:**
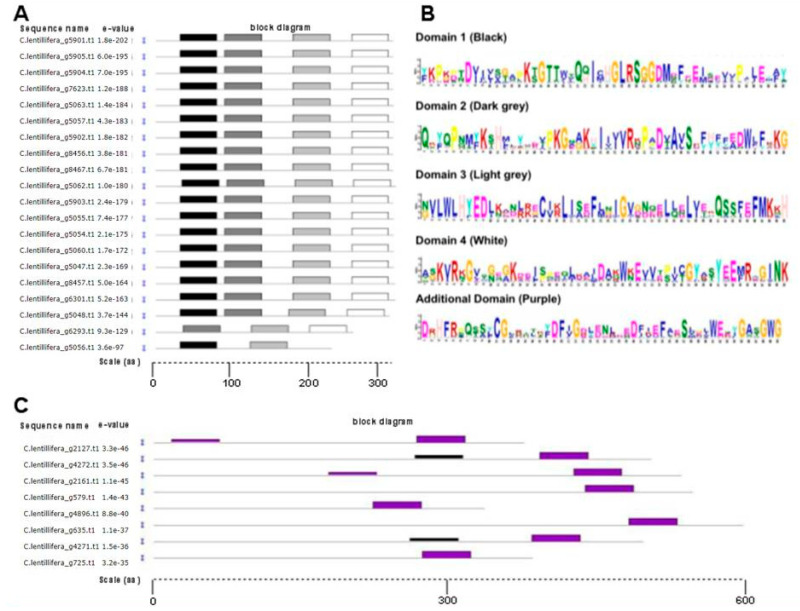
(**A**) Analysis of the typical four conserved domains conserved of 20 *Caulerpa* sulfotransferases; canonical motifs were indicated by black, dark grey, light grey and white blocks. (**B**) Consensus sequences of the identified domains. (**C**) Conserved motif analysis of 8 *Caulerpa* sulfotransferases containing peculiar domains, purple blocks indicate the presence of the additional domain.

Conventionally, sulfotransferases are characterized by four conserved domains [48,55,56,57]. In order to investigate canonical domains in *Caulerpa* proteins, a conserved domain analysis was made using the MEME bioinformatic tools (Figure 5). This analysis indicated the presence in *C*. *lentillifera* of 20 sulfotransferases containing the four conserved domains (Figure 5A,B). Among these, g6293.t1 showed the absence of the conserved domain 1, while g5056.t1 showed the absence of the conserved domains 3 and 4. Domain 1 is characterized by the presence of the motif KT/SGTTWXG, necessary for PAPs binding [55], domain 2 showed the presence of the catalytic histidine [46], and domain 4 showed the motif KYRXG. The other 17 proteins showed no or less conserved sequences among the canonical domains. Among these, eight sulfotransferases (namely, g579.t1, g635.t1, g725.t1 g2127.t1, g2161.t1, g4271.t1, g4272.t1, g4896.t1) present an additional conserved domain (Figure 5C). This domain is located at C-terminal, with the exception of g2127.t1 and g2161.t1, showing a double domain.

Another classification of sulfotransferases is based on their subcellular localization, discriminating between membrane-associated proteins and cytosolic isoforms. The former are responsible for sulfation of biopolimers, peptides, sulfoconjugation of steroids and other natural products [52,57]. In silico prediction of putative transmembrane sulfotransferase identified 11 proteins with transmembrane domains or transit peptide (Table 4). As expected, the 18 *Caulerpa* sulfotransferases orthologous with the cytosolic *At*SOT18 showed no plasma membrane localization signal.

Among the plasma membrane sulfotransferase, g1228, g2821.t1, g3179.t1 and g8270.t1 and g579.t1, g635.t1, g4173.t1, g4271.t1 and g4272.t1 showed similar positions in two clusters of the phylogenetic tree (Figure 2). Interestingly, Delos et al. [58] showed a plasma membrane localization for the human *Hs*3ST2 sulfotrasferase which is involved in the uncommon 3-O-sulfation of the heparan sulfate. Accordingly, a similar function can be predicted by structure analysis of g1228.t1 and g4176.t1. Contrarily, tyrosil sulfotrasferases are usually located in plasma membrane [46,57]. This is in contrast with the absence of transmembrane and transit peptide domains reported for g1631.t1. 

### 2.4. Expression Analysis Revealed Tissue Specificity for Sulfotransferases from Caulerpa

To our knowledge, only two studies have been published about RNA-seq expression analysis on *Caulerpa* sp. so far [59,60]. Specifically, a comprehensive expression atlas of the algal tissues, namely apex, pinnules, rachis, bases, stolons and holdfast in *Caulerpa taxifolia* has been reported by Ranjan et al. [59]. In order to increase our knowledge, we mined the *C*. *taxifolia* transcriptome using the *C*. *lentillifera* sulfotransferase sequence in order to identify possible orthologous. It is worth pointing out that no evaluation about sulfotransferases roles—and their molecular expression—was previously argued on *C*. *taxifolia*. Particularly, the entire set of sulfotransferases has not been identified yet, thus highlighting a necessary demand for a complete elucidation in other *Caulerpa* specie(s) used for -omic approach.

Firstly, we identified the best similar hits, finding only 14 transcripts (Supplemental Table S2), then we selected every transcript showing a query coverage (QC) ≥ 40% and identity (I) ≥ 50%. Each selected transcript showed an alignment e-value comprised between 1.28e-97 and 9.33e-58. This analysis identified 57 putative transcripts; among these, only 25 were previously identified as sulfotransferase, 15 transcripts were annotated as different proteins and 17 transcripts were not annotated yet. In order to confirm the identification of the 57 *C*. *taxifolia* transcripts, a BLASTx approach analysis confirmed the annotation as sulfotransferase for 40 sequences. The 17 “fake” sulfotransferases have not been utilized in further analyses. Considering that the dataset by Ranjan et al. [59] derived by RNA sequencing, it is not possible discriminate the number of genes and the number of alternative transcripts. Reasonably, it is worth presuming a similar gene duplication of the sulfotransferase family also in *C*. *taxifolia*.

We report an expression analysis of *Caulerpa* sulfotransferases by using the expression atlas by Ranjan et al. [59], (Table 5). Generally, sulfotransferases are mainly expressed in pinnae and rachis. These two tissues showed a high number—about 16 and 15, respectively—of strongly expressed transcripts (reads count ≥ 100). Frond apex showed 11 high expressed transcripts while the other tissues showed 7 high expressed transcripts. Actually, the identified transcripts showed lower expression abundance in the basal part of the algae: stolon and holdfast. Accordingly, Arimoto et al. [60] reported an enrichment of GO categories related to “Starch binding” and “Carbohydrate binding” comparing gene expressions of frond and stolons, thus suggesting an increased carbohydrate metabolism in the upper part of the algae. Furthermore, 7 sulfotransferases were ubiquitously expressed in all tissues. A similar, interesting parallel has been reported in the brown algae *Saccharina japonica* [61,62]. In fact, genes related to the expression of mannitol, alginate and fucoidans showed a tissue specific expression, reflecting in some cases their accumulation in specific tissues. Generally, *Caulerpa* sulfotransferase showed an expression pattern similar among tissues, but differences were observed in expression values. Ctaxi_contig_27571 and Ctaxi_contig_16182 represented exceptions. The first showed a peculiar and strong expression in pinnae and rachis, while the second was expressed only in frond bases and rachis.

The higher number (14) of transcripts with absence or poor FPKM (≤50) counts in all tissues suggests the presence of a number of pseudogenes showing no, or an unknown, role in *Caulerpa*. Computational mistakes during the transcriptome assembly cannot be excluded. Alternatively, it is possible that the physiological growth conditions used by Ranjan et al. [59] are not adequate to induce the expression of these genes. Specific transcriptional regulation of sulfotransferase in different algae was reported by various authors. The development stage, perturbing conditions, and light are examples of factors inducing differential expression of sulfotransferase in green algae, brown algae and diatoms [53,63,64,65]. Finally, it could be suggested a tissue-specific localization of metabolic processes, independent by the peculiar multinucleated cell which composed *Caulerpa*.

A global pattern of specific transcriptional expression polar distributed from holdfast to apex was suggested [59,60]. In this view, a genetic regulation flow started with DNA regulation in stolons, passed through rachis with mRNA translation, resulting in protein accumulation in the apex [59]. Accordingly, metabolic and physiologic regulations appeared to be focused on fronds. This hypothesis is sustained by the presence of phytoregulator-related genes for the synthesis of ABA, auxin, cytochinin, brassinosteroids and others [60]. Consistently, Raman spectroscopy of wound plugs of *C*. *taxifolia* revealed site-specific chemical gradients for β-carotene and caulerpenyne [66]. Particularly, caulerpenyne derivates were transformed in active compounds by enzymatic modifications occurred through different tissue sub-zones and finally recruited in the wounding site [66]. Under this view a site-specific biosynthesis of sulfated polysaccharides, in the active parts of *Caulerpa*, as well as other important biocompounds, could be an effective and peculiar mechanism of physiological and metabolic regulation of this alga.

### 2.5. Reconstruction of the Sulfated Polysaccharides Pathway

Sulfated polysaccharides are particularly represented among the *Ulvophyceae* as a marine ecosystem adaptation. Depending on the sugar composition of the SPs, this algae class can be divided in two different groups [67]. The first group showed uronic acid-rich polysaccharides, the second, uronic acid-limited polysaccharides. The latter includes *Codium*, *Bryopsis* and *Caulerpa* [68]. Recently, several research groups characterized SPs from *Caulerpa*, defining a sugar SPs structure composed by a galactomannan backbone and the presence of xylose and rhamnose [19,31,32,33].

**Figure 6:**
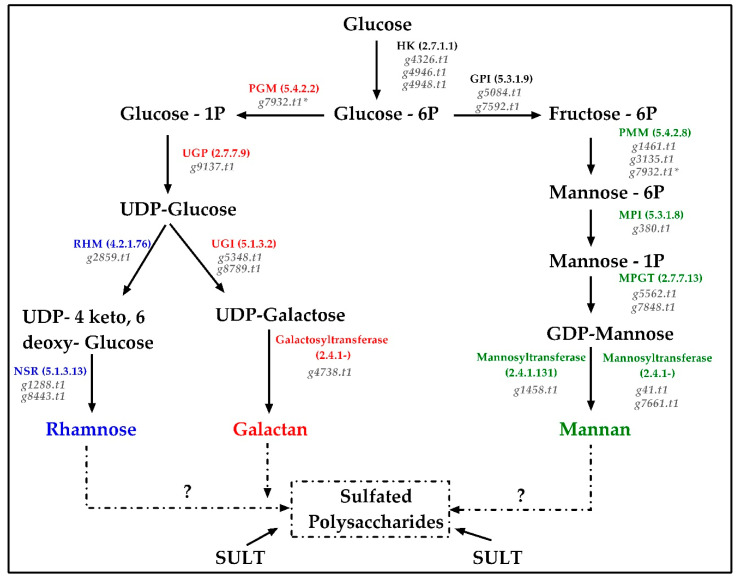
Scheme of proposed SPs biosynthetic pathway in *Caulerpa*. Legend: HK = hexokinase; GPI = glucose-6-phosphate isomerase; PMM = phosphoglumutase; MPI = mannose-phosphate isomerase; MPGT = mannose-1-phosphate guanylyltransferase; PGM = phosphoglucomutase; UGP = UTP-glucose-1-phosphate urydyltransferase; UGI = UDP glucose isomerase; RHM = UDP-4-keto-L-rhamnose-reductase; NSR = dTDP-4-dehydrorhamnose reductase; SULT = sulfotransferase. * = g7932.t1 was annotated as bifunctional PMM/PGM.

In order to define a putative SPs biosynthetic pathway of *Caulerpa*, we mined the genome to identify those genes able to build a galactomannan polysaccharide (Figure 6). We reported the presence of genes required to obtain GDP-Mannose and UDP-Galactose. Interestingly, g7932 is bifunctional phosphomanno/glucomutase. Alignment similarity was identified with Esi_0149_0031 of the brown alga *E*. *silicolusus*.

In order to identify genes coding for enzymes synthetizing mannans and galactans, we selected annotated proteins with the predicted E.C. number 2.4.1.*n*. *Caulerpa* genome presents nine different genes: g41.t1, g137.t1, g4748.t1, g5003.t1, g6758.t1, g643.t1, g934.t1, g7661.t1 and g1485.t1. Using a BLASTp approach, we selected g41.t1 and g7661.t1 as suitable mannosyltransferases and g4748.t1 as galactosyltransferase. The other genes are glycotransferases involved in sulfolipid sulfoquinovosyl diacylglycerol (SQDG) biosynthesis (g6758.t1 and g1485.t1), glycoprotein biosynthesis (g643.t1) and glycosyl inositol biosynthesis (g934.t1). In addition, g1458 (E.C. 2.4.1.131) was reported as α1,2-mannosyltransferase, while four different genes were identified as xylogalactosyltransferase (not reported in Figure 6): g5792.t1 (E.C. 2.4.1.133); g3840.t1, g5551.t1 and g5992.t1 (E.C. 2.4.1.134). Furthermore, five genes were annotated as mannan endo-1,4-beta-mannosidase (E.C. 3.2.1.78): g1178.t1, g5650.t1, g6715.t1, g7172.t1 and g8306.t1. These genes are involved in the degradation of the mannan unit.

Comparison with Rhodophyta (*C*. *crispus*) and brown algae (*E*. *silicolosus*) has been performed in order to ascertain possible convergent pathways. Similarities were identified for the initial steps of the pathway represented by ubiquitous enzymes shared by algae, plants and animals. For example, phosphoglumutase (PMM) and mannose-phosphate isomerase (MPI) showed similarities with CHC_T00009219001 (*Cc*PMM) and CHC_T00005574001 (*Cc*MPI). Curiously, *Caulerpa* MPI (g380.t1) presented similarities with *Ec*MPI 2-3-4 (Esi_0000_0207, Esi_0120_0009, Esi_0120_0005) but differences with Esi_0195_0002 (*Ec*MPI-1). As expected, no hit was reported in the *Caulerpa* genome for fucosyltransferase, mannuronan C-5-epimerase and mannunoran synthase, which are involved in fucoidans and alginate synthesis in *E*. *silicolosus* [51]. Analogous results were obtained using k-carrageenases and chondroitin synthase, which are responsible for the biosynthesis in red algae SPs [48].

Rhamnose biosynthetic related genes were identified using *Arabidopsis thaliana* genes *At*1g53500 (RHM—E.C. 4.2.1.76) and *At*1g63000 (NSR/ER—5.1.3.13). The first identified g2859.t1 showing a query coverage (QC) of 50% and identities (I) about 63% while the second identifying g1288.t1 and g8443.t1 showing QC = 93%; I = 48 and QC = 94; I = 50, respectively. Interestingly, the enzyme coding by *At*1g63000 is the first eukaryotic reported gene able to catalyze both rhamnose synthase, reductase and epimerase activities [69].

Plant galactomannan biosynthesis required three different genes to catalyze the final step of the pathway: mannan synthase (MANs), galactomannan-galactosyl transferase (GMGT) and α-galactosidase [70]. Using sequences from different plants we identified two different putative MANs in *Caulerpa* genome: g267.t1 and g3897.t1. These proteins were originally annotated as glucomannan 4-beta-mannosyltransferase and showed a ≥90% of QC and ≥38% I compared with *A*. *thaliana* and *Coffea arabica* mannan synthase protein sequences. These results suggest a possible role of these two *Caulerpa* enzymes in galactomannan synthesis. Contrarily, GMGT comparison identified one sequence (g5317.t1) showing poor alignment stats vs. GMGT plant sequences and α-galactosidase analysis retrieved no hit. In detail, g5317.t1 showed a ≤30% QC and ≤30% I compared with *Coffea canephora*, *C*. *arabica* and *Lotus japonicas* while showing a 60% QC and 25% I compared with *Vitis viniferae*. Prediction analysis on g5317.t1 showed ambiguous results about a hypothetical galactosyltransferase function. As a whole, these results suggest probable peculiarities in the final step of galactomannans backbone biosynthesis in *Caulerpa*. Considering the comparisons between *Caulerpa vs* red/brown-algae and plants, it is possible assume the existence of unknown mannosyl- and galactosyl-transferase mechanisms for the biosynthesis of the final polysaccharide backbone (Figure 6). Putative candidates for the final reactions could be g267.t1, g3897.t1 and g5317.t1, together with undiscovered enzymatic players. Furthermore, a role of mannan endo-1,4-beta-mannosidases should be no excluded in biosynthesis processes in addition to polysaccharides catabolism.

Finally, orthologous genes of the galactomannan biosynthesis pathway were identified in *C*. *taxifolia* (Supplemental Table S3–S4). Each analyzed reaction showed in Figure 6, reported one expressed gene, at least. As for sulfotransferase, these genes showed a higher reads-count in pinnae and rachis.

## 3. Materials and Methods

### 3.1. Algae Genome Scan

Identification of sulfotransferase in *Caulerpa lentillifera* was obtained by selecting specific PFAM domains (PF00685, PF03567, PF06990 and PF0084), using the OIST marine database at https://marinegenomics.oist.jp/umibudo/viewer?project_id = 55.

Identification of sulfotransferase from different algal species was obtained using the same PFAM domains at the algal genomics resource database at https://phycocosm.jgi.doe.gov selecting *Chlamydomonas reinhardtii*, *Dunaliella salina*, *Volvox carteri*, *Ostreococcus tauri*, *Thalassiosira pseudonana*, *Phaeodactylum tricornutum*, *Ectocarpus siliculosus*, *Fragilariopsis cylindrus*, *Nannochloropsis oceanic*, *Cyanidioschyzon merolae* and *Chondrus crispus*. PFAM domains were selected as indicated in the PFAM database related manuscript paper [47].

### 3.2. Phylogenetic Analysis

Sequences of *Caulerpa* sulfotransferase were found using the OIST marine database at https://marinegenomics.oist.jp/umibudo/viewer?project_id = 55. Other algae and plants sequences were found using uniprot database (https://www.uniprot.org/) and TAIR database. Alignments and phylogenetic analyses were done using the software MEGA version 6 [71]. Sequence alignments were obtained using the MUSCLE algorithm. The alignments were performed using Gap penalties: Gap Open = -0.01, Gap extend = 0, Hydrophobicity Multiplier = 1.2. The number of max iterations was equal to 64. The substitution model was selected by using a ProtTest approach to select the best-fit models of protein evolution using MEGA [72]. The used test tree was a neighbor-joining tree and the statistical method was Maximum Likelihood. Models with the lowest BIC scores (Bayesian Information Criterion) were considered as the best to describe the substitution pattern. The phylogenetic tree was constructed using the maximum likelihood method with the LG gamma distributed substitution model. Tree inference options were set by Nearest-Neighbor-Interchange (NNI) method. The test of phylogeny was performed using the bootstrap method with a number of bootstrap replication equal to 100.

### 3.3. Structures and Localization Prediction

Transmembrane prediction was performed using TOPCONS online software http://topcons.cbr.su.se/ [73] and TMHMM server 2.0 http://www.cbs.dtu.dk/services/TMHMM/.

Structure prediction was performed using the protein homology/analogy recognition engine software v 2.0 (Phyre2) at http://www.sbg.bio.ic.ac.uk/~phyre2/html/page.cgi?id = index [74]. Conserved motif analysis was performed by the Multiple Em for Motif Elicitation (MEMESuite4.11.1) server 5 [75]. The analysis was performed using the classic optimizes discovery mode, setting the possibility of site distribution occurrence from zero to one at least per sequence. The minimum and maximum motif width was set to 10 and 50, respectively, while the maximum number of motifs was defined as four.

### 3.4. BLAST Approaches and Expression Analysis

Blast approaches were conducted using the Geneious software version 2020.1.1 by the use of a temporary license [76]. Customized databases were constructed using the FASTA supplementary files from the *C*. *lentillifera* genome [1] and from the *C*. *taxifolia* transcriptome [59]. The sulfotransferases protein sequences obtained by the *Caulerpa lentillifera* genome scan (Materials and Methods 4.1), and putative protein sequences related to the SPs biosynthetic pathway were used to identify orthologous sequences in *C*. *taxifolia* using a tBLASTn approach. BLAST parameters were matrix = BLOSUM62, gap cost = 11.1, max e-value = 10; adjustment = conditional compositional score matrix adjustment method. Transcripts were considered a good sulfotransferase candidate when they showed a query coverage (QC) ≥ 40% and identity (I) ≥ 50%. BLASTx approach vs. the NCBI database was used to confirm the putative sulfotransferase annotation. Sequences with disaccording annotated function were not considered yet. The selected transcripts showed an alignment e-value comprised between 1.28e-97 and 9.33e-58.

An additional TBLASTn approach was performed using protein sequences of SPs biosynthetic enzymes from brown, red, green algae and plants vs. the *C*. *taxifolia* and *C*. *lentillifera* customized databases. This analysis was performed in order to clarify possibly unknown passages of the *Caulerpa* SPs biosynthetic pathway. TBLASTn parameters were matrix = BLOSUM62, gap cost = 11.1, max e-value = 10; adjustment = conditional compositional score matrix adjustment method.

Expression analysis was obtained by the transcriptomic atlas published by Ranjan et al. [59]. As described by the authors RNA-seq was performed on 4–5 different samples from different tissues (frond apex, rachis, pinnules, frond base, stolon and holdfast) of *C*. *taxifolia* and RSEM was used to obtain normalized counts [59,77].

## 4. Conclusions

In conclusion, the results of these extensive bioinformatic investigations indicate unique characteristics observed among both sulfotransferase and SPs biosynthetic enzymes in *Caulerpa*. Particularly, glycosyltranferases catalyzing the final steps of the galactomannan synthesis showed differences compared with the well-characterized glycosyltranferases in plants and algae.

These results provide useful information for the selection of *Caulerpa* species for both commercial applications, and genetic studies to improve the synthesis of valuable products.

Further studies in silico and experimental data on biochemical, physiological and molecular properties of these enzymes are required for a better understanding of the biosynthetic pathways and to improve information for the exploitation of *Caulerpa* metabolites.

## Figures and Tables

**Table 1:** List of beneficial actions proposed for bio-compounds and extract from different *Caulerpa* species.

Specie	Proposed Beneficial Effects	Bio-Compounds	References
*Caulerpa cupressoides*	Antinociceptive and anti-inflammatory effects	Lectin	[35]
	Antioxidant and antiurolithic effects	Sulfated polysaccharides	[20]
	Immunostimulatory activity	Sulfated polysaccharides	[34]
	Possible anticoagulant and antioxidant effects	Sulfated polysaccharides	[31]
*Caulerpa cylindracea*	Antioxidant and antimicrobial activity	Fatty acid derived products	[4]
	Anxiolytic effect in *Diplodus sargus*	Caulerpin	[38]
	Regulation of oxidative phosphorylation and of AMPKα1 pathway in cancer cells	Caulerpin	[39]
*Caulerpa lentillifera*	Anti-inflammatory activity	Sulfated polysaccharides	[19]
	Anticoagulant properties	Not specified	[40]
	Antimicoytic activity	Not specified	[41]
	Possible immunomodulator roles	Sulfated polysaccharides	[33]
*Caulerpa mexicana*	Gastroprotective effects (reduction of ethanol damage)	Sulfated polysaccharides	[36]
*Caulerpa okamurae*	Anti-inflammatory; increases of insulin sensitivity in adipocytes and macrophages	Not specified	[26]
*Caulerpa prolifera*	Osteogenic potential	Sulfated polysaccharides	[37]
*Caulerpa racemosa*	Antinociceptive and anti-inflammatory effects	Sulfated polysaccharides	[30]
	Antioxidant and antibacterial activity	Different compounds	[27]
	Immunostimulatory activity (increased of macrophages, no cytokines induction)	Polysaccharides	[32]
	Positive effects on murine colon damages	Caulerpin	[42]
	Radical scavenging activities	Not specified	[29]
	Reduction of pain in the rat temporomandibular joint	Sulfated polysaccharides	[18]
	Therapeutic role in breast cancer	Racemosin	[43]
*Caulerpa scalpelliformis*	Non-specific immunity and disease resistance in fish (*Nile tilapia*)	Not specified	[44]
*Caulerpa sertularioides*	Antibacteric activity vs. *V*. *parahaemolyticus* and *V*. *alginolyticus*	Not specified	[45]
*Caulerpa Sp*	Antiaeging and UV protection action on mice	Not specified	[28]

**Table 2:** List of identified sulfotransferase and sulfates in *C*. *lentillifera* genome. Redundant hit for PF00685 and PF13469 were underlined. Significance and domain position were indicated in Supplemental Table S1.

PF00685 (1)		PF00685 (2)		PF03567		PF06990		PF00884	
Sulfotransferase		Sulfotransferase		Sulfotransferase		Sulfotransferase		Sulfatase	
Proteins ID	Best BLASTp Hit	Proteins ID	Best BLASTp Hit	Protein ID	Best BLASTp Hit	Proteins ID	Best BLASTp Hit	Proteins ID	Best BLASTp Hit
g1228.t1	Sulfotransferase	g5063.t1	Sulfotransferase	g395.t1	ABC transporter	No hit		g5709.t1	Sulf-hydrolase/transferase
g1262.t1	Glutamyl endopeptidase	g5901.t1	Sulfotransferase	g579.t1	Sulfotransferase			g6993.t1	Sulf-hydrolase/transferase
g1631.t1	Sulfotransferase like	g5902.t1	Sulfotransferase	g635.t1	Sulfotransferase				
g1631.t2	Sulfotransferase like	g5902.t2	Sulfotransferase	g725.t1	Sulfotransferase				
g2821.t1	Sulfotransferase	g5903.t1	Sulfotransferase	g2127.t1	Sulfotransferase				
g3147.t1	Peptidyl-prolyl cis-trans isomerase	g5904.t1	Sulfotransferase	g2161.t1	Sulfotransferase				
g3179.t1	Sulfotransferase	g5905.t1	Sulfotransferase	g3703.t1	Acetyl-CoA carboxylase				
g4176.t1	Sulfotransferase	g6293.t1	Sulfotransferase	g3783.t1	Serine acetyltransferase				
g5047.t1	Sulfotransferase	g6301.t1	Sulfotransferase	g4170.t1	Cytochrome p450				
g5048.t1	Sulfotransferase	g7623.t1	Sulfotransferase	g4173.t1	Sulfotransferase				
g5054.t1	Sulfotransferase	g8270.t1	Sulfotransferase	g4271.t1	Sulfotransferase				
g5055.t1	Sulfotransferase	g8456.t1	Sulfotransferase	g4272.t1	Sulfotransferase				
g5056.t1	Sulfotransferase	g8457.t1	Sulfotransferase	g4272.t2	Sulfotransferase				
g5057.t1	Sulfotransferase	g8467.t1	Sulfotransferase	g4896.t1	Sulfotransferase				
g5060.t1	Sulfotransferase								
g5062.t1	Sulfotransferase								

**Table 3:** Putative function of sulfotransferases from *C*. *lentillifera* obtained by structures similarity. Co = coverage; Cf = confidence.

Predicted Structures Similarity	*C*. *lentillifera* Proteins
Heparan sulfate glucosamine 3-o-sulfotransferase	g1228.t1 (Co = 56%; Cf = 100%); g4176.t1 (Co = 31%; Cf = 100%)
Protein-tyrosine sulfotransferase 2;	g1631.t1 (Co = 50%; Cf = 100%)
Heparan sulfate n-deacetylase/n-sulfotransferase	g2821.t1 (Co = 47%; Cf = 100%); g3179.t1 (Co = 39%; Cf = 100%)
Sulphotransferase-18 from Arabidopsis	g5047.t1; g5048.t1; g5054.t1; g5055.t1; g5056.t1; g5057.t1; g5060.t1; g5062.t1; g5063.t1; g5901.t1; g5902.t1; g5903.t1; g5904.t1; g5905.t1; g6293.t1; g6301.t1; g7623.t1; g8467.t1 (Co = 81–87%; Cf = 100%)
Haparan sulfate n-deacetylase/n-2 sulfotransferase	g8270.t1 (Co = 56%; Cf = 100%).
Human sulfotranferase sult1c3 in complex with pap	g8456.t1 (Co = 82%; Cf = 100%); g8457.t1 (Co = 82%; Cf = 100%)
Maltose-binding periplasmic protein, heparan sulfate 2-o	g579.t1 (Co = 99%; Cf = 43%); g725.t1 (Co = 99%; Cf = 51%); g4271.t1 (Co = 98%; Cf = 41%); g4896.t1 (Co = 98%; Cf = 62%).
Maltose binding protein - heparan sulfate 6-o	g635.t1 (Co = 99%; Cf = 33%); g4173.t1 (Co = 58%; Cf = 99.3%); g4272.t1 (Co = 99%; Cf = 37%)
Human pregnenolone sulfotransferase	g2127.t1 (Co = 54%; Cf = 99%); g2161.t1 (Co = 43%; Cf = 99%)
N-acetylgalactosamine-6-sulfatase	g5709.t1 (Co = 70%; Cf = 100%); g6993.t1 (Co = 69%; Cf = 100%)

**Table 4:** Putative identification of transmembrane sulfotransferase from *C*. *lentillifera*. TM = Transmembrane; TP = Transit peptide, P = Prediction probability.

Gene Id	TM	Number TM	TM Position (aa)	TP	TP Position (aa)
g579.t1	No	0	-	Yes	5–24 (P = 100%)
g635.t1	No	0	-	Yes	7–27 (P = 100%)
g1228.t1	Yes	1	415–434 (P = 100%)	Yes	1–23 (P = 40%)
g2161.t1	Yes	1	7–26	Yes	6–26 (P = 100%)
g2821.t1	Yes	1	476–498 (P = 100%)	Yes	1–31 (P = 20%)
g3179.t1	Yes	2	7–29; 581–603 (P = 100%)	Yes	1–23 (P = 100%)
g4173.t1	Yes	1	13–41 (P = 100%)	No	-
g4176.t1	Yes	2	309–329 (P = 40%); 765–787 (P = 100%)	No	-
g4271.t1	No	0	-	Yes	1–26 (P = 100%)
g4272.t1	Yes	1	13–33 (P = 100%)	No	-
g8270.t1	Yes	1	418–440 (P = 100%)	Yes	1–22 (P = 20%)

**Table 5:** RNA-seq of *C*. *taxifolia* sulfotransferae in different tissues. Expression data were obtained using the dataset by Ranjan et al. [59]. Colors indicate the degree of expression (lower expression: red to higher expression: green).

	RSEM Read Counts					
*C. taxifolia* Transcripts	Frond Apex	Frond Base	Holdfast	Pinnae	Rachis	Stolon
Ctaxi_contig_10628|comp31456_c3_seq1	44.4	22.0	16.0	28.3	42.2	22.5
Ctaxi_contig_10917|comp31547_c2_seq1	107.0	40.0	30.9	83.2	90.1	61.0
Ctaxi_contig_10918|comp31547_c2_seq2	44.6	14.5	49.0	34.8	46.7	49.4
Ctaxi_contig_15098|comp32496_c3_seq2	61.0	68.0	41.0	115.4	145.6	45.6
Ctaxi_contig_15401|comp32550_c1_seq1	54.2	46.0	47.6	54.0	73.7	57.4
Ctaxi_contig_15402|comp32550_c1_seq2	1.4	3.4	2.7	5.4	5.9	7.0
Ctaxi_contig_16178|comp32679_c5_seq7	23.7	15.2	8.0	41.2	44.5	16.1
Ctaxi_contig_16179|comp32679_c5_seq8	56.4	35.9	40.0	68.2	55.0	70.5
Ctaxi_contig_16180|comp32679_c5_seq9	20.6	15.6	18.8	38.0	33.5	18.3
Ctaxi_contig_16182|comp32679_c5_seq11	77.1	23.9	23.4	17.8	70.8	38.5
Ctaxi_contig_16717|comp32777_c2_seq1	5.2	3.4	2.5	10.1	12.4	1.8
Ctaxi_contig_18258|comp33042_c0_seq1	92.7	60.1	64.5	97.9	76.9	108.4
Ctaxi_contig_18292|comp33050_c1_seq1	1879.0	1765.0	595.6	5246.9	4964.1	907.1
Ctaxi_contig_23234|comp33768_c0_seq2	62.2	70.8	57.5	113.9	125.6	55.4
Ctaxi_contig_23235|comp33768_c1_seq1	86.2	64.4	57.6	136.1	147.3	41.0
Ctaxi_contig_24623|comp33959_c2_seq1	237.4	157.8	241.0	247.0	230.9	242.5
Ctaxi_contig_24659|comp33966_c1_seq3	86.4	55.6	65.9	122.3	115.2	80.2
Ctaxi_contig_24664|comp33966_c1_seq8	27.6	27.2	19.6	39.7	42.0	23.6
Ctaxi_contig_24669|comp33966_c1_seq13	29.8	21.0	20.5	42.5	38.1	20.4
Ctaxi_contig_26858|comp34188_c2_seq1	20.6	19.1	18.6	49.4	34.5	22.3
Ctaxi_contig_26859|comp34188_c2_seq2	84.7	66.2	90.9	124.0	119.3	97.2
Ctaxi_contig_27564|comp34267_c0_seq1	202.3	136.6	106.5	428.3	310.6	136.4
Ctaxi_contig_27571|comp34267_c3_seq1	47.7	40.2	12.0	130.6	85.4	19.0
Ctaxi_contig_40054|comp35467_c2_seq1	715.5	595.7	444.6	1266.3	1549.7	549.7
Ctaxi_contig_40059|comp35467_c3_seq1	172.8	107.2	117.2	197.4	167.2	86.5
Ctaxi_contig_47488|comp35975_c4_seq1	62.8	30.4	36.5	56.4	40.9	26.9
Ctaxi_contig_47489|comp35975_c4_seq2	360.6	209.4	251.1	452.0	455.4	211.7
Ctaxi_contig_47490|comp35975_c4_seq3	0.0	0.0	1.0	0.2	0.0	0.0
Ctaxi_contig_47491|comp35975_c4_seq4	3.6	0.0	2.6	3.4	3.1	0.4
Ctaxi_contig_47493|comp35975_c4_seq6	9.6	3.4	2.9	4.9	2.8	2.2
Ctaxi_contig_47494|comp35975_c4_seq7	0.1	0.0	0.1	0.2	0.1	0.1
Ctaxi_contig_47495|comp35975_c4_seq8	143.8	68.9	66.5	142.1	144.5	64.6
Ctaxi_contig_47496|comp35975_c4_seq9	1.7	2.0	3.1	8.0	4.4	3.4
Ctaxi_contig_47497|comp35975_c4_seq10	468.7	442.3	308.2	632.8	643.2	499.7
Ctaxi_contig_47499|comp35975_c4_seq12	121.7	93.5	77.8	161.7	174.7	87.6
Ctaxi_contig_47500|comp35975_c4_seq13	46.4	35.1	30.5	49.8	61.4	38.7
Ctaxi_contig_56778|comp36555_c3_seq5	141.0	49.2	47.5	84.9	79.4	62.6
Ctaxi_contig_70773|comp37223_c1_seq6	97.2	81.5	59.5	181.2	128.2	73.0
Ctaxi_contig_9279|comp31033_c1_seq1	13.8	7.1	4.8	10.0	11.2	8.9
Ctaxi_contig_9848|comp31227_c0_seq3	25.7	24.6	16.3	45.7	41.9	22.6

## Supplementary Materials

The following are available online at https://www.mdpi.com/1422-0067/21/18/6681/s1.
