# Supplementary material for: Bioinformatic Characterization of Sulfotransferase Provides New Insights for the Exploitation of Sulfated Polysaccharides in Caulerpa
Source: Int J Mol Sci. 2020 Sep 12;21(18):6681. doi: 10.3390/ijms21186681 (PMC7554865; doi:10.3390/ijms21186681)
Supplement: Supplementary file 1 [file ijms-21-06681-s001.pdf]

International Journal of *Molecular Sciences*

MDPI

Supplemental material

Bioinformatic characterization of sulfotransferase provides new insights for the exploitation of sulfated polysaccharides in *Caulerpa*

Simone Landi, Sergio Esposito

Supplemental Figure S1. Alignment of g1631.t1 and g1631.t2

```
C.lentilifera_g1631.t1      ------------------------------------------------------------
C.lentilifera_g1631.t2      MMQFVLTLKLRERNELCFGHFEIMILCRNVVFTFVPLLLHFALADVIRISELDGVVGGIR

C.lentilifera_g1631.t1      ----------MLFVKLGIALQEIDQLHPDGRKRLPEAEDAYRLALQLKMSAEIRVMVMGN
C.lentilifera_g1631.t2      KRILDGDRNPMLFVKLGIALQEIDQLHPDGRKRLPEAEDAYRLALQLKMSAEIRVMVMGN
                                      **************************************************

C.lentilifera_g1631.t1      LAVLLMSSNRVYEAIKVMQECIQITQRDRLSVKNITGTLFNYGKALSILGQVNEAENAYL
C.lentilifera_g1631.t2      LAVLLMSSNRVYEAIKVMQECIQITQRDRLSVKNITGTLFNYGKALSILGQVNEAENAYL
                            ************************************************************

C.lentilifera_g1631.t1      EVLIQSRGVDAKNFAKAFASLKHFPKELETEVQCIGNYVLHGSIDSIKCNSSAASWWDHF
C.lentilifera_g1631.t2      EVLIQSRGVDAKNFAKAFASLKHFPKELETEVQCIGNYVLHGSIDSIKCNSSAASWWDHF
                            ************************************************************

C.lentilifera_g1631.t1      SLEDKSWILFAAFHSLQNAQDDLSIHQSWLYISMANELQNELLLKSAFPASLFSVKSGLS
C.lentilifera_g1631.t2      SLEDKSWILFAAFHSLQNAQDDLSIHQSWLYISMANELQNELLLKSAFPASLFSVKSGLS
                            ************************************************************

C.lentilifera_g1631.t1      DPTPIFIVGMPRSGSTLLEQALASHPGVFALGEDTPFAPLVPKIIEEFHKSSPADLSVIG
C.lentilifera_g1631.t2      DPTPIFIVGMPRSGSTLLEQALASHPGVFALGEDTPFAPLVPKIIEEFHKSSPADLSVIG
                            ************************************************************

C.lentilifera_g1631.t1      QEYIDEVRKQIPSGMKPIRTVDKMLNNVLNLGFVELTLPSACLLYITRHPMDCALSCYLQ
C.lentilifera_g1631.t2      QEYIDEVRKQIPSGMKPIRTVDKMLNNVLNLGFVELTLPSACLLYITRHPMDCALSCYLQ
                            ************************************************************

C.lentilifera_g1631.t1      PFEGRGTPWAWTLHNIGERYRLIYKLQQHWDRVMPDKVLTVYYEKLVWNFEHEMRRVLKH
C.lentilifera_g1631.t2      PFEGRGTPWAWTLHNIGERYRLIYKLQQHWDRVMPDKVLTVYYEKLVWNFEHEMRRVLKH
                            ************************************************************

C.lentilifera_g1631.t1      CGLQWEDSILQFYKNDRAVLTASSTQVRQELFRTSIGRWKKYAKYLQPLNDVLGDIVDEY
C.lentilifera_g1631.t2      CGLQWEDSILQFYKNDRAVLTASSTQVRQELFRTSIGRWKKYAKYLQPLNDVLGDIVDEY
                            ************************************************************

C.lentilifera_g1631.t1      EHRLRELDVSHNEL
C.lentilifera_g1631.t2      EHRLRELDVSHNEL
                            **************
```

Supplemental Figure S2. Alignment of g4272.t1 and g4272.t2.

```
C.lentilifera_g4272.t1      MINSSKRFQRRRCGNLLLIIFVLCVGTLVLFNLNEKSTLRLS--GKSSLSYSSQSILSTS
C.lentilifera_g4272.t2      MINSSKRFQRRRCGNLLLIIFVLCVGTLVLFNLNEKSTLRLSALGKSSLSYSSQSILSTS
                            ******************************************  ****************

C.lentilifera_g4272.t1      SNTMDKKTGSAAWSDDIPTFYIYDVFLRGEEMLSLRGECEASLPIEFCTSLFQSKSRRSR
C.lentilifera_g4272.t2      SNTMDKKTGSAAWSDDIPTFYIYDVFLRGEEMLSLRGECEASLPIEFCTSLFQSKSRRSR
                            ************************************************************

C.lentilifera_g4272.t1      KDEAQLYLVPIFEPVSIDRESTVDAIERLRSDRYFLKFAGSNFIFVCYSSNCQSTARAVH
C.lentilifera_g4272.t2      KDEAQLYLVPIFEPVSIDRESTVDAIERLRSDRYFLKFAGSNFIFVCYSSNCQSTARAVH
                            ************************************************************

C.lentilifera_g4272.t1      YALTKVNRSIWISRNDTINWPCRKRVINVDEIHRSIGLSSEADRESHALSQSLKEAGKLI
C.lentilifera_g4272.t2      YALTKVNRSIWISRNDTINWPCRKRVINVDEIHRSIGLSSEADRESHALSQSLKEAGKLI
                            ************************************************************

C.lentilifera_g4272.t1      EGRNRWRCKGHSKWGTNVTRIFDFGTLKDTSFKSQSNKVAYCGVPKVGSSVTVLMMRRMN
C.lentilifera_g4272.t2      EGRNRWRCKGHSKWGTNVTRIFDFGTLKDTSFKSQSNKVAYCGVPKVGSSVTVLMMRRMN
                            ************************************************************

C.lentilifera_g4272.t1      GMPDWKLANTVDIRNFKTGYKWRYPNGHNTLQMYDSTDWVKGMMVRNPITRLLSGYRSKI
C.lentilifera_g4272.t2      GMPDWKLANTVDIRNFKTGYKWRYPNGHNTLQMYDSTDWVKGMMVRNPITRLLSGYRSKI
                            ************************************************************

C.lentilifera_g4272.t1      EDLREFSRIPGGWHKTHPPSFEEFVQTIVDKEATGTIDWIDRHFRPQSALCGVRTLSYDF
C.lentilifera_g4272.t2      EDLREFSRIPGGWHKTHPPSFEEFVQTIVDKEATGTIDWIDRHFRPQSALCGVRTLSYDF
                            ************************************************************

C.lentilifera_g4272.t1      IGRYENRAQDIKEFLESLELWESIGATGWGVNETGAIFSQDEQIVRKNKPKPVTHADSKS
C.lentilifera_g4272.t2      IGRYENRAQDIKEFLESLELWESIGATGWGVNETGAIFSQDEQIVRKNKPKPVTHADSKS
                            ************************************************************

C.lentilifera_g4272.t1      MIQQYYTEDLIKTVMDLYNEDFNRFGFSKNIDDYL
C.lentilifera_g4272.t2      MIQQYYTEDLIKTVMDLYNEDFNRFGFSKNIDDYL
                            ***********************************
```

Supplemental Figure S3. Alignment of g5902.t1 and g5902.t2.

```
C.lentilifera_g5902.t1      MEVLEQPPKGVKKLSDNLRTLIPPEATRMAFEYKPKDTDIMVVGTPKTGTTWIQHIMHGL
C.lentilifera_g5902.t2      MEVLEQPPKGVKKLSDNLRTLIPPEATRMAFEYKPKDTDIMVVGTPKTGTTWIQHIMHGL
                            ************************************************************

C.lentilifera_g5902.t1      RSGGDMEFEEMSETVPILEYFYKRPGYDITTPQTYPPNMFKTHVVHDDATIGNAKRIMIV
C.lentilifera_g5902.t2      RSGGDMEFEEMSETVPILEYFYKRPGYDITTPQTYPPNMFKTHVVHDDATIGNAKRIMIV
                            ************************************************************

C.lentilifera_g5902.t1      RDPADVAVSKFYYFENWLYNEGEMTLEEFVEWCFLKPCPPYDIYWNIVDMNFIVQAYPHR
C.lentilifera_g5902.t2      RDPADVAVSKFYYFENWLYNEGEMTLEEFVEWCFLKPCPPYDIYWNIVDMNFIVQAYPHR
                            ************************************************************

C.lentilifera_g5902.t1      KDEGVLWLHYEDLKADLKGCIKLISDFMGIGVGNQDLLNLIEHQSSFDFMKQHKDKFDTG
C.lentilifera_g5902.t2      KDEGVLWLHYEDLKADLKGCIKLISDFMGIGVGNQDLLNLIEHQVRMYHI----------
                            ********************************************  : .:

C.lentilifera_g5902.t1      FVKKLTKSELEITSEEEYKKSASKIRKGVIGEGKKEISPEQLKALDAKWKEVVEPICGYA
C.lentilifera_g5902.t2      ------------------------------------------------------------


C.lentilifera_g5902.t1      SYDEMRTSINKELRRTF
C.lentilifera_g5902.t2      TYESLG-----------
                            :*:.:
```

International Journal of Molecular Sciences

MDPI

Supplemental Table S1. List of identified of sequences from C. lentillifera genome containing sulfotransferase and sulfatase related domains (PF00685, PF13469 and PF03567).

Proteins ID	PFAM domain 1	Domain position	e-value	PFAM domain 2	domain position	e-value
g1228.t1	PF00685	71-360	3.4e-08	PF13469	68-111	3.3e-13
g1262.t1	PF00685	60-294	3.0e-16	PF13469	60-206	1.3e-14
g1631.t1	PF00685	302 522	1.1e-12	PF13469	304-487	1.8e-35
g2821.t1	PF00685	168-354	6.5e-08	PF13469	253-338	1.6e-10
g3147.t1	PF00685	204-249	2.5e-12	PF13469	202-230	6.4e-11
g3179.t1	PF00685	65-329	4.5e-09	PF13469	157-177	1.4e-06
g4176.t1	PF00685	370-639	1.2e-11	PF13469	368-498	3.0e-11
g5047.t1	PF00685	37-286	1.5e-27			
g5048.t1	PF00685	37-280	2.0e-19			
g5054.t1	PF00685	38-286	3.5e-27			
g5055.t1	PF00685	37-237	1.8e-28			
g5056.t1	PF00685	38-181	1.6e-09			
g5057.t1	PF00685	37-238	1.0e-26			
g5060.t1	PF00685	37-244	2.1e-25			
g5062.t1	PF00685	40-238	1.9e-26			
g5063.t1	PF00685	37-233	1.7e-25			
g5901.t1	PF00685	37-291	2.4e-31			
g5902.t1	PF00685	37-223	6.2e-26	PF13469	77-210	7.7e-10
g5903.t1	PF00685	37-291	3.6e-32	PF13469	92-208	4.7e-09
g5904.t1	PF00685	37-290	2.0e-33	PF13469	78-210	9.1e-09
g5905.t1	PF00685	37-288	3.5e-30	PF13469	63-210	8.9e-09

g6293.t1	PF00685	40-231	3.2e-24			
g6301.t1	PF00685	38-287	5.4e-30	PF13469	113-210	1.8e-08
g7623.t1	PF00685	37-286	3.9e-31			
g8270.t1	PF00685	64-299	5.4e-07	PF13469	267-302	6.3e-11
g8456.t1	PF00685	37-290	1.5e-33	PF13469	113-210	3.4e-06
g8457.t1	PF00685	38-287	7.8e-33	PF13469	98-213	2.2e-09
g8467.t1	PF00685	37-289	5.0e-32	PF13469	113-210	8.1e-06
g395.t1	PF03567	146-380	7.8e-09			
g579.t1	PF03567	319-545	1.9e-18			
g635.t1	PF03567	358-590	7.7e-22			
g725.t1	PF03567	150-377	1.2e-23			
g2127.t1	PF03567	149-371	4.5e-19			
g2161.t1	PF03567	311-533	6.5e-17			
g3703.t1	PF03567	49-152	4.2e-06			
g3783.t1	PF03567	147-384	1.9e-07			
g4170.t1	PF03567	146-371	1.1e-09			
g4173.t1	PF03567	132-356	6.5e-07			
g4271.t1	PF03567	268-497	9.9e-23			
g4272.t1	PF03567	275-507	3.9e-25 8			
g4896.t1	PF03567	97-333	3.2e-24			
g5709.t1	PF00884	1-287	0.0			
g6993.t1	PF00884	1-288	0.0			

Supplemental Table S2. Comparison between sulfotransferase proteins in *C.lentillifera* and *C. taxifolia* transcripts obtained using a tBlastn approach.

	Caulerpa taxifolia				
C. lentillifera	**Gene**	**Query coverage (%)**	**Identities (%)**	**Positivies (%)**	**eValue**
g1228.t1	Ctaxi_contig_24623 | comp33959_c2_seq1	92.1	59.6	77.5	1.65e-76
g1631.t1	Ctaxi_contig_40412 | comp35500_c0_seq1	25.8	68	85.6	9.33e-58
g2821.t1	Ctaxi_contig_23235 | comp33768_c1_seq1	53.1	54.3	74.2	7.11e-104
g3179.t1	Ctaxi_contig_71149 | comp37240_c1_seq10	60.8	40.8	57.4	2.11e-89
g4176.t1	Ctaxi_contig_23235 | comp33768_c1_seq1	33.1	59.2	81.1	5.98e-121
g5047.t1	Ctaxi_contig_47489 | comp35975_c4_seq2	66.3	70.3	84.4	1.84e-111
g5048.t1	Ctaxi_contig_47489 | comp35975_c4_seq2	66.9	64.2	64.2	2.90e-102
g5054.t1	Ctaxi_contig_47489 | comp35975_c4_seq2	67.9	66.7	66.7	1.33e-105
g5055.t1	Ctaxi_contig_47489 | comp35975_c4_seq2	67.6	68.8	82.8	5.59e-111
g5056.t1	Ctaxi_contig_10917 | comp31547_c2_seq1	54	64.1	77.9	2.30e-33
g5057.t1	Ctaxi_contig_47489 | comp35975_c4_seq2	67.9	74.1	85.6	5.50e-113
g5060.t1	Ctaxi_contig_47489 | comp35975_c4_seq2	67.1	73.5	87	6.69e-116
g5062.t1	Ctaxi_contig_47489 | comp35975_c4_seq2	66.9	68.8	80.9	1.35e-109
g5063.t1	Ctaxi_contig_47489 | comp35975_c4_seq2	67.9	71.3	85.2	1.26e-115
g5901.t1	Ctaxi_contig_24659 | comp33966_c1_seq3	53.6	71.2	85.3	5.05e-62
g5902.t1	Ctaxi_contig_24659 | comp33966_c1_seq3	53.9	70.2	85.4	6.65e-91
g5902.t2	Ctaxi_contig_15401 | comp32550_c1_seq1	65.6	53.3	75.3	1.71e-58
g5903.t1	Ctaxi_contig_24659 | comp33966_c1_seq3	53.6	67.6	89.4	1.89e-89
g5904.t1	Ctaxi_contig_24659 | comp33966_c1_seq3	53.6	70.6	87.6	2.02e-92
g5905.t1	Ctaxi_contig_24659 | comp33966_c1_seq3	53.6	67.1	86.5	1.81e-87
g6293.t1	Ctaxi_contig_15401 | comp32550_c1_seq1	57.5	73.0	86.2	4.61e-82
g6301.t1	Ctaxi_contig_15401 | comp32550_c1_seq1	47.1	80.1	88.1	8.28e-89
g7623.t1	Ctaxi_contig_24659 | comp33966_c1_seq3	53.6	68.2	82.9	2.41e-80
g8270.t1	Ctaxi_contig_24623 | comp33959_c2_seq1	84.3	55.2	71.4	1.48e-153
g8456.t1	Ctaxi_contig_24659 | comp33966_c1_seq3	53.6	64.7	81.2	2.05e-80
g8457.t1	Ctaxi_contig_24659 | comp33966_c1_seq3	53.4	60.8	83.6	2.00e-81

g8467.t1	Ctaxi_contig_24659 | comp33966_c1_seq3	53.6	61.8	81.2	5.78e-78
g579.t1	Ctaxi_contig_69968 | comp37187_c0_seq3	76.2	38.3	54.7	8.33e-73
g635.t1	Ctaxi_contig_69968 | comp37187_c0_seq3	64.8	37.0	57.2	5.18e-89
g725.t1	Ctaxi_contig_69966 | comp37187_c0_seq1	99.2	58.1	76.8	6.46e-176
g2127.t1	Ctaxi_contig_10628 | comp31456_c3_seq1	49.7	77	84.8	1.17e-104
g2161.t1	Ctaxi_contig_69968 | comp37187_c0_seq3	87.5	35.1	55	1.28e-97
g4173.t1	Ctaxi_contig_56778 | comp36555_c3_seq5	43.7	78.9	88.2	2.41e-92
g4271.t1	Ctaxi_contig_40059 | comp35467_c3_seq1	47.5	66.3	84.4	5.67e-121
g4272.t1	Ctaxi_contig_40059 | comp35467_c3_seq1	47.3	78.6	90.5	2.94e-145
g4272.t2	Ctaxi_contig_40059 | comp35467_c3_seq1	47.1	78.6	90.5	2.53e-145
g4896.t1	Ctaxi_contig_40059 | comp35467_c3_seq1	70.9	64	79.8	2.65e-113
g5709.t1	Ctaxi_contig_15098 | comp32496_c3_seq2	39.4	71.6	85.1	6.88e-106
g6993.t1	Ctaxi_contig_15098 | comp32496_c3_seq2	39.6	73	87	6.17e-106

Supplemental Table S3. Comparison between sulfated polysaccharides related proteins in *C. lentillifera* and *C. taxifolia* transcripts obtained using a tBlastn approach. QC = Query coverage; I=identities.

C. lentillifera	*C. taxifolia*	QC	I	e-value
g1458.t1	Ctaxi_contig_39338|comp35404_c3_seq1	30.49%	84.7%	1.19e-68
g5792.t1	Ctaxi_contig_27056|comp34208_c1_seq1	48.19%	89.2%	4.39e-122
g5992	Ctaxi_contig_60160|comp36743_c0_seq1	27.82%	81.1%	6.65e-78
g3840	Ctaxi_contig_48338|comp36032_c1_seq3	37.41%	93.2%	5.21e-92
g5551.t1	Ctaxi_contig_51463|comp36240_c2_seq3	45.78%	89.3%	9.66e-107
g4738.t1	Ctaxi_contig_30931|comp34635_c0_seq4	30.31%	85.1%	6.95e-92
g3897	Ctaxi_contig_48555|comp36047_c1_seq8	49.29%	78.4%	5.12e-147
g267	Ctaxi_contig_48553|comp36047_c1_seq6	23.81%	77.9%	1.25e-134
g5084	Ctaxi_contig_48568|comp36048_c0_seq7	34.62%	96.1%	4.26e-125
g7592	Ctaxi_contig_7624|comp30243_c1_seq1	55.67%	56.8%	3.86e-140
g4326	Ctaxi_contig_47346|comp35967_c2_seq3	84.89%	53.8%	1.04e-91
g4946	Ctaxi_contig_47346|comp35967_c2_seq3	94.50%	68.9%	4.87e-155
g4948	Ctaxi_contig_50614|comp36185_c0_seq2	48.39%	50.0%	6.59e-38
g41	Ctaxi_contig_11828|comp31795_c0_seq1	62.50%	92.4%	9.43e-79
g7661.t1	Ctaxi_contig_66433|comp37048_c0_seq1	25.99%	70.3%	3.05e-71
g5572	Ctaxi_contig_31740|comp34719_c1_seq1	82.39%	86.3%	4.38e-154
g7868	Ctaxi_contig_31740|comp34719_c1_seq1	68.89%	83.5%	2.01e-136
g380	Ctaxi_contig_20320|comp33350_c1_seq2	81.48%	73.5%	1.53e-118
g1228	Ctaxi_contig_19435|comp33202_c2_seq12	37.94%	66.7%	2.61e-43
g8443	Ctaxi_contig_19424|comp33202_c2_seq1	59.52%	78.7%	7.71e-86
g3135	Ctaxi_contig_12132|comp31865_c0_seq1	66.60%	76.1%	2.45e-98
g1461	Ctaxi_contig_67885|comp37105_c3_seq1	51.25%	84.6%	5.37e-72
g7932	Ctaxi_contig_15692|comp32599_c0_seq5	31.06%	96.4%	5.40e-136
g2859	Ctaxi_contig_27946|comp34311_c0_seq8	46.83%	92.4%	7.73e-118

g5348	Ctaxi_contig_14229|comp32325_c0_seq1	86.01%	92.2%	4.49e-39
g8789	Ctaxi_contig_14230|comp32325_c0_seq2	76.23%	87.8%	6.72e-40
g9137	Ctaxi_contig_33402|comp34869_c1_seq1	28.30%	25.3%	6.77e-01

International Journal of *Molecular Sciences*

MDPI

Supplemental Table S4. Expression analysis of Polysaccharides biosynthesis enzymes in *C. lentillifera* and *C. taxifolia* in different tissues. Expression data were obtained using the dataset by Ranjan et al. [2015].

E.C. number	C. lentillifera	C. taxifolia	Frond Apex	Frond Base	Holdfast	Pinnae	Rachis	Stolon
2.4.1.131	g1458.t1	Ctaxi_contig_39338|comp35404_c3_seq1	11,974	5,768	8,075	14,506	15,674	11
2.4.1.133	g5792.t1	Ctaxi_contig_27056|comp34208_c1_seq1	2633,842	2932,674	2234,66	2515,16	3477,774	5879,502
2.4.1.134	g5992	Ctaxi_contig_60160|comp36743_c0_seq1	90	50,4	45,0325	66,996	71,994	50,2
2.4.1.134	g3840	Ctaxi_contig_48338|comp36032_c1_seq3	179,624	87,194	51,4225	196,546	210,31	131,838
2.4.1.134	g5551.t1	Ctaxi_contig_51463|comp36240_c2_seq3	218,876	276,01	234,7475	680,592	704	299,178
2.4.1.-	g4738.t1	Ctaxi_contig_30931|comp34635_c0_seq4	336,764	261,432	278,81	396,686	436,75	270,808
2.4.1.32	g3897	Ctaxi_contig_48555|comp36047_c1_seq8	197,966	142,96	150,905	228,946	235,742	153,338
2.4.1.32	g267	Ctaxi_contig_48553|comp36047_c1_seq6	173,006	89,028	125,5275	234,462	184,172	140,272
5.3.1.9	g5084	Ctaxi_contig_48568|comp36048_c0_seq7	128,252	136,494	86,1425	212,96	245,748	134,546
5.3.1.9	g7592	Ctaxi_contig_7624|comp30243_c1_seq1	0	2	1	5,2	1,6	0,2
2.7.1.1	g4326	Ctaxi_contig_47346|comp35967_c2_seq3	354,72	329,444	150,0275	494,054	430,224	316,36
2.7.1.1	g4946	Ctaxi_contig_47346|comp35967_c2_seq3	261,328	235,208	128,78	354,804	351,94	240,394
2.7.1.1	g4948	Ctaxi_contig_50614|comp36185_c0_seq2	47,046	15,19	16,13	54,436	58,658	22,978
2.4.1.-	g41	Ctaxi_contig_11828|comp31795_c0_seq1	257,6	145,198	109,405	250,048	277,654	160,4
2.4.1.-	g7661.t1	Ctaxi_contig_66433|comp37048_c0_seq1	50,4	21,976	23,5	52,8	40,61	29,018
2.7.7.13	g5572	Ctaxi_contig_31740|comp34719_c1_seq1	236,416	232,696	198,9575	462,848	428,21	292,888
2.7.7.13	g7868	Ctaxi_contig_31740|comp34719_c1_seq1	236,416	232,696	198,9575	462,848	428,21	292,888
5.3.1.8	g380	Ctaxi_contig_20320|comp33350_c1_seq2	62,842	21,934	29,275	65,034	46,282	21,782
5.1.3.13	g1228	Ctaxi_contig_19435|comp33202_c2_seq12	85,592	124,272	148,12	140,176	162,088	148,86
5.1.3.13	g8443	Ctaxi_contig_19424|comp33202_c2_seq1	36,182	91,97	92,0475	67,446	86,758	68,782
5.4.2.8	g3135	Ctaxi_contig_12132|comp31865_c0_seq1	34,862	74,866	63,65	94,82	75,546	102,374
5.4.2.8	g1461	Ctaxi_contig_67885|comp37105_c3_seq1	52,114	47,6	41,73	116,402	92,252	74,882

5.4.2.2	g7932	Ctaxi_contig_15692|comp32599_c0_seq5	193,928	113,234	106,7475	153,14	270,012	151,798
4.2.1.76	g2859	Ctaxi_contig_27946|comp34311_c0_seq8	131,2	120,212	150,7475	157,976	181,93	207,958
5.1.3.2	g5348	Ctaxi_contig_14229|comp32325_c0_seq1	389,552	177,472	276,0575	324,642	378,084	301,2
5.1.3.2	g8789	Ctaxi_contig_14230|comp32325_c0_seq2	447,13	95,254	113,4525	164,38	234,656	187,2
2.7.7.9	g9137	Ctaxi_contig_33402|comp34869_c1_seq1	531,704	480,21	442,0925	577,412	735,99	385,04
